# Embryogenic competence of microspores is associated with their ability to form a callosic, osmoprotective subintinal layer

**DOI:** 10.1093/jxb/ery458

**Published:** 2019-01-31

**Authors:** Alba Rivas-Sendra, Patricia Corral-Martínez, Rosa Porcel, Carolina Camacho-Fernández, Antonio Calabuig-Serna, Jose M Seguí-Simarro

**Affiliations:** Cell Biology Group - COMAV Institute, Universitat Politècnica de València (UPV), Valencia, Spain

**Keywords:** Androgenesis, *Brassica napus*, calcium, cellulose, cell wall, doubled haploids, eggplant, rapeseed

## Abstract

Microspore embryogenesis is an experimental morphogenic pathway with important applications in basic research and applied plant breeding, but its genetic, cellular, and molecular bases are poorly understood. We applied a multidisciplinary approach using confocal and electron microscopy, detection of Ca^2+^, callose, and cellulose, treatments with caffeine, digitonin, and endosidin7, morphometry, qPCR, osmometry, and viability assays in order to study the dynamics of cell wall formation during embryogenesis induction in a high-response rapeseed (*Brassica napus*) line and two recalcitrant rapeseed and eggplant (*Solanum melongena*) lines. Formation of a callose-rich subintinal layer (SL) was common to microspore embryogenesis in the different genotypes. However, this process was directly related to embryogenic response, being greater in high-response genotypes. A link could be established between Ca^2+^ influx, abnormal callose/cellulose deposition, and the genotype-specific embryogenic competence. Callose deposition in inner walls and SLs are independent processes, regulated by different callose synthases. Viability and control of internal osmolality are also related to SL formation. In summary, we identified one of the causes of recalcitrance to embryogenesis induction: a reduced or absent protective SL. In responding genotypes, SLs are markers for changes in cell fate and serve as osmoprotective barriers to increase viability in imbalanced *in vitro* environments. Genotype-specific differences relate to different responses against abiotic (heat/osmotic) stresses.

## Teaser

A reduced, genotype-dependent ability to form a protective subintinal layer that serves as osmoprotective barrier to increase viability is identified as a source of recalcitrance to induction of microspore embryogenesis.

## Introduction

Microspore embryogenesis is an experimental system whereby embryogenic development is induced in cells initially programmed to become male gametophytes. Through the application of specific abiotic stresses, haploid microspores are induced to exit their original developmental program and to produce microspore-derived embryos (MDEs) and eventually plants, which may be either haploid or doubled-haploid (DH). This inducible morphogenic pathway provides a fascinating system to study plant cell totipotency and the mechanisms underlying it. In addition, DH technology is a very powerful tool to produce pure lines for hybrid seed production. However, DH technology is still far from possible in a number of important but recalcitrant crops. Thus, advances in our understanding of this developmental switch may help us to improve our knowledge of the molecular and cellular basis of totipotency and morphogenesis, and to overcome technical limitations for DH production in recalcitrant crops. Embryogenic microspore divisions follow a pattern and mechanism (Parra-Vega *et al.*, 2015) more similar to somatic-type cytokinesis than to asymmetric first pollen division. In somatic-type cytokinesis, the first polysaccharides to form new cell plates are pectins and hemicelluloses transported via Golgi-derived vesicles, whereas callose is synthesized *in situ* (reviewed by Drakakaki, 2015). At this stage, callose-rich walls are discontinuous, flexible, and delicate. Transient callose walls serve as stabilizing scaffolds to expand the cell plate prior to cellulose deposition and callose removal by β-1,3-glucanases (reviewed by Seguí-Simarro *et al.*, 2008). The precise timing of the first cellulose deposition still remains controversial (Chen *et al.*, 2018), but it seems clear that it must be preceded by callose removal (Verma, 2001), so that by the end of cytokinesis, the primary wall is formed by a cellulose, hemicellulose, and pectin network (Chen *et al.*, 2018), and callose is only present around plasmodesmata. In contrast, embryogenic microspores produce walls with altered polysaccharide composition. In rapeseed (*Brassica napus*), cellulose is absent from the first cell walls (Dubas *et al.*, 2014), whilst the presence of callose extends over time, producing transient callose-rich and cellulose-defective walls (Parra-Vega *et al.*, 2015). In parallel, a subintinal layer (SL) is formed between the intine and the plasma membrane, even before the first embryogenic division is observed. SLs are variable in thickness, but entirely surround cells during the first embryogenic stages. SLs were found exclusively in dividing microspores, being absent in pollen-like structures exposed to the same culture conditions. This has led to the notion that the presence of a SL is an early marker of embryogenic commitment (Parra-Vega *et al.*, 2015).

The stress used to induce embryogenesis in many species, including rapeseed and eggplant (*Solanum melongena*), is heat. In general, the way cells perceive heat involves transient increases in Ca^2+^ mediated by changes in plasma membrane fluidity and activation of Ca^2+^-permeable channels (Saidi *et al.*, 2009). The signaling cascade initiated by heat-induced Ca^2+^ influx acts as inducer of many components of the heat-shock response, but the effects of Ca^2+^ as an intracellular messenger go far beyond that. Ca^2+^ plays a key regulatory role in many different processes (White and Broadley, 2003). For example, Furch *et al.*, (2007) showed that heat shock applied to leaves is transmitted to sieve elements, where the release of Ca^2+^ into the lumen induces transient callose deposition. Another example is the induction of microspore embryogenesis. Modification of plasma membrane properties such as fluidity and permeability and increases in the cytosolic Ca^2+^ concentration are acknowledged as necessary prerequisites to trigger abiotic stress-induced callose synthesis (Stass and Horst, 2009). These prerequisites are present in the induction of microspore embryogenesis, where heat shock produces changes in membrane fluidity (Dubas *et al.*, 2013) and transient increases in Ca^2+^ levels (Rivas-Sendra *et al.*, 2017*a*). Indeed, a relationship between Ca^2+^ and microspore embryogenesis has been long suspected (Reynolds, 1990, 2000; Cho and Kasha, 1995; Hoekstra *et al.*, 1997; Pauls *et al.*, 2006), but it has never been explored in depth.

Plant cells store Ca^2+^ in different compartments such as the endoplasmic reticulum, vacuoles, and cell walls, where it is tightly bound to pectin. Ca^2+^ plays a key role in cell wall formation and regulation of specific callose synthase (CalS) complexes (Verma and Hong, 2001). Twelve different CalS genes have been described in *Arabidopsis thaliana* (Hong *et al.*, 2001), and functional homologs have been found in species such as rice (Shi *et al.*, 2014), grapevine (Yu *et al.*, 2016), tomato (Adkar-Purushothama *et al.*, 2015), tobacco (Cai *et al.*, 2011), maize (Slewinski *et al.*, 2012), citrus (Enrique *et al.*, 2011), and rapeseed (Sun *et al.*, 2017). In Arabidopsis, these genes are indistinctly named *CalS* (from callose synthase) or *GLS* (from *GLUCAN SYNTHASE-LIKE*). In rapeseed, more than 50 genes, including several duplicates, have been found as orthologous to Arabidopsis *CalS* genes. They are divided into 11 groups and named after their Arabidopsis counterparts, except for *CalS4/GSL9* (Sun *et al.*, 2017). Among the different Arabidopsis CalS genes, five are found in microspores/pollen. *CalS11/GSL1* and *CalS12/GSL5* are present during all stages of pollen development, but their main function is the synthesis of tetrad callose walls after meiosis (Enns *et al.*, 2005). *CalS5/GSL2* forms a thin callose layer in young microspores, which is needed as scaffold for proper exine deposition and patterning (Dong *et al.*, 2005). CalS5/GSL2 presents a Ca^2+^-binding domain in the central cytoplasmic hydrophilic loop and is also involved in callose deposition during pollen-tube growth (Dong *et al.*, 2005). However, it has been shown that such deposition does not need Ca^2+^, which has been interpreted as CalS5/GSL2 being Ca^2+^-independent, at least for this process (Schlüpmann *et al.*, 1993). *CalS9/GSL10* and *CalS10/GSL8* are responsible for the formation of the callose wall that separates generative and vegetative cells after the first pollen mitosis (Töller *et al.*, 2008).

Given the links between heat stress, alteration of membrane fluidity, increases in Ca^2+^, callose synthesis by CalSs, tolerance to osmotic stress, and microspore embryogenesis, it has been speculated that Ca^2+^ could be involved in the formation of the unusual SL and inner walls that define the first stages of microspore embryogenesis (Parra-Vega *et al.*, 2015). To examine this, in this work we studied cell wall and SL formation, including the dynamics of callose and cellulose deposition, and intracellular Ca^2+^ during microspore embryogenesis in a model system, the highly embryogenic rapeseed DH4079 line. To determine whether these processes are related to embryogenic competence, we also studied two recalcitrant lines, one of rapeseed (DH12075) and one of eggplant (DH36). In order to explore the relationship between Ca^2+^ and callose and cellulose synthesis, we used several compounds known to interfere with intracellular Ca^2+^ levels, plasma membrane fluidity, and/or callose synthesis and deposition. These compounds included caffeine, digitonin, and endosidin 7 (ES7). Caffeine inhibits cell plate formation during plant cytokinesis by reducing membrane-associated Ca^2+^ (Paul and Goff, 1973), thereby inhibiting callose synthesis (Samuels and Staehelin, 1996). Digitonin is a detergent known to increase membrane permeability, Ca^2+^ uptake, and subsequent callose synthesis even more strongly and more specifically than the ionophore A23187 (Waldmann *et al.*, 1988). ES7 inhibits callose deposition specifically during somatic-type cytokinesis and during pollen-tube growth, but not in other processes such as the pathogen response in physically wounded tissues and the formation of sieve plates (Park *et al.*, 2014).

To correlate the observed phenotypes to the activity of specific enzymes, we assessed the expression of rapeseed CalS genes. Among the five CalSs found during microspore and pollen development, we focused on *CalS5/GSL2*, *CalS9/GSL10*, and *CalS10/GSL8*, which are reported to be active in vacuolated microspores and young pollen, the stages used to induce embryogenesis (Rivas-Sendra *et al.*, 2015). In addition, we studied the expression of *CalS12/GSL5*, which is also involved in callose deposition upon wounding and in the pathogen response (PR). Interestingly, several enzymes involved in PR, including chitinases, have been found to be strongly up-regulated upon stress treatment in barley anther cultures (Jacquard *et al.*, 2009), and two chitinase isoforms are excreted by developing maize MDEs (Borderies *et al.*, 2004). These coincidences between PR and microspore embryogenesis made us think that *CalS12/GSL5* could be another PR-related gene involved in microspore embryogenesis. Finally, we examined changes of internal and external osmolality of cultured microspores at different stages, to check whether it has a role on embryogenic competence, as suggested in different legume species (Grewal *et al.*, 2009; Ochatt *et al.*, 2009). Together, our results shed light on the relationship between Ca^2+^ levels and callose deposition, and establish a functional link between them and increased viability upon induction of microspore embryogenesis.

## Materials and methods

### Plant materials

Rapeseed (*Brassica napus*) cv. Topas lines DH4079 and DH12075 donor plants were grown in chambers at COMAV (Universitat Politècnica de València, Spain) and Wageningen University and Research Centre (Wageningen, The Netherlands). Plants were grown in 20-cm pots at 60% humidity and a 16/8 h photoperiod, at 20 °C until flowering and then at 10 °C. Eggplant (*Solanum melongena*) line DH36 (Rivas-Sendra *et al.*, 2017*b*) donor plants were grown in 30-cm pots under natural light in greenhouses at COMAV.

### Microspore cultures

Isolation, induction treatment, and culturing of rapeseed microspores were performed according to Custers (2003). We considered three bud-length groups, 3.0–3.2, 3.3–3.4, and 3.5–3.6 mm, which typically cover the embryonic-responsive stages and some younger and/or older stages. Buds were measured and sorted into these three groups, which were processed in parallel. After estimating culture responses by counting cell divisions, the best-responding group was assumed to contain the most microspores/pollen at the responsive stages. Buds of each length group were dissected, surface-sterilized with 2% NaClO (10 min), washed three times in sterile dH_2_O (15 min), and crushed with a sterile syringe piston in NLN medium (Nitsch and Nitsch, 1967) supplemented with 13% sucrose and filter-sterilized. Microspores were isolated by filtration through 30-μm nylon mesh (Millipore) followed by three rounds of centrifugation at 800 rpm (4 min each) in a refrigerated Eppendorf Centrifuge 5804R with a 17.5-cm rotor radius. Microspore density was adjusted to 4×10^4^ microspores ml^–1^. Suspensions were plated, incubated in darkness for 24 h at 32.5 °C for DH4079 and 33 °C for DH12075 to induce embryogenesis, and then at 25 °C in darkness for progression of embryogenesis.

Eggplant microspore culture was performed according to Rivas-Sendra *et al.*, (2017*b*). Anthers containing mostly vacuolate microspores were dissected, surface-sterilized with 70% ethanol (30 s) and with 4 g l^–1^ NaClO (5 min), and rinsed three times in sterile dH_2_O. Anthers were crushed in sterile dH_2_O, microspores were filtrated through a 41-µm nylon mesh, then retained in and released from a 11-µm nylon mesh, and finally centrifuged three times at 800 rpm (4 min each). Isolated microspores were resuspended in sterile dH_2_O at 5×10^5^ microspores ml^–1^, plated and incubated at 35 °C in darkness for 3 d to induce embryogenesis. After the induction treatment, microspores were collected, centrifuged, and resuspended as described above in NLN medium plus 2% sucrose, 0.5 mg l^–1^ 1-naphthaleneacetic acid, and 0.5 mg l^–1^ 6-benzilaminopurine, and incubated at 25 °C in darkness for progression of embryogenesis.

### Detection of Ca^2+^, callose, and cellulose

For detection of Ca^2+^, microspore cultures were collected in conical tubes, centrifuged at 100 *g* for 4 min at room temperature, resuspended in PBS, mixed with the same volume of 0.2 g l–1 FluoForte (Enzo Life Sciences, ENZ-52015) in PBS, and incubated in darkness (30 min). Cells were then washed with PBS, centrifuged at 200 *g* (2 min), mounted on microscope slides with Mowiol anti-fading mounting solution (17% Mowiol 4–88 from Sigma-Aldrich +33% glycerol, v/v, in PBS) and observed immediately.

For detection of callose and cellulose, rapeseed microspore cultures were collected at days 3 and 6 after isolation, fixed overnight at 4 °C with 4% paraformaldehyde in PBS (pH 7.4), washed three times with PBS, and stored at 4 °C in 0.1% paraformaldehyde in PBS until use. Before staining, samples were immobilized by mixing 10 μl of microspore pellets with the same volume of 1.8% agarose and letting it solidify on a microscope slide. For detection of callose, fixed samples were first counterstained by incubating them with 10 µg ml^–1^ propidium iodide (PI; Fluka) in PBS (10 min), then they were washed three times with PBS, stained with 0.1% aniline blue (AB; Fluka) in PBS (20 min), washed three times with the same buffer, and mounted with Mowiol mounting medium and then observed. For eggplant DH36 microspores, detection of callose with AB was unsuccessful because of interference of the signal with exine autofluorescence that was impossible to eliminate, thus making it impossible to obtain clear, informative confocal images. Therefore, detection of callose was carried out by immunogold labeling in plastic-embedded samples (see below). For detection of cellulose, fixed samples were stained with 0.01% Pontamine Fast Scarlet (S4B) in 0.1 M PBS for 30 min (Anderson *et al.*, 2010), washed three times with PBS, mounted with a 1:1 mix of Mowiol mounting medium and 2.5 μg ml^–1^ DAPI (Sigma-Aldrich), and prepared as described in Custers (2003) for nuclei staining, and incubated for at least 15 min. All incubations were performed in darkness.

For Ca^2+^, callose, and cellulose, a minimum of 20 cells of each species, each stage, and each developmental fate were examined at 40× magnification. Samples were observed and imaged using a Leica CTR 5500 and a Zeiss LSM 780 confocal laser-scanning microscopes. Digital images were processed using the Leica Application Suite Advanced Fluorescence (LAS AF) and Fiji software.

### Chemical treatments

Caffeine was added directly to the culture medium prior to sterilization. The other chemicals were diluted from previously prepared stock solutions: digitonin (1 g l^–1^) was prepared in distilled water, ES7 (5 mM and 20 mM) were prepared in DMSO. All chemicals were purchased from Sigma-Aldrich except for ES7 (Hit2Lead). The stock solutions were filter-sterilized and stored at –20 °C. Corresponding volumes of each stock solution were added to the microspore cultures to reach the desired final working concentration, as explained in the Results section. Three different, replicated experiments were performed with at least three culture dishes per experiment for each chemical tested, and at least 20 different individual structures per stage were observed.

### Processing and immunogold labeling for electron microscopy

Samples of rapeseed and eggplant microspore cultures were collected at equivalent development stages (days 3 and 9 after isolation, respectively), and processed according to Parra-Vega *et al.* (2015) and Corral-Martínez *et al.* (2016). Microspores were concentrated by centrifugation, cryoprotected with 20% dextran, transferred to aluminum sample holders, and high-pressure frozen in a Leica HPM100 system (Leica Microsystems). Samples were then freeze-substituted in 2% OsO_4_ in anhydrous acetone at –80 °C (4 d), followed by slow warming to room temperature over a period of 24 h. After rinsing in several acetone washes, they were removed from their holders and infiltrated with increasing concentrations of Spurr resin (Ted Pella, Redding, CA) in acetone according to the following schedule: 4 h in 2% and then 5% resin, 12 h in 10%, 25%, 50%, and 75% resin, and 40 h in 100% resin. Polymerization was performed at 70 °C for 30 h. Using a Leica UC6 ultramicrotome, thin sections (1 μm) were obtained for light microscopy, and ultrathin sections (~80 nm) were obtained for electron microscopy.

For anti-callose immunogold labeling, ultrathin sections were mounted on Formvar and carbon-coated 150-mesh nickel grids, then hydrated with dH_2_O (1 min), with PBS (1 min), blocked with 5% BSA in PBS (5 min), and incubated at 25 °C (1 h) with anti-callose monoclonal antibody (Biosupplies, Australia) diluted 1:5000 in 1% BSA. Sections were then washed three times with PBS and incubated at 25 °C (45 min) with a goat anti-mouse secondary antibody conjugated with 10-nm colloidal gold (BBI Solutions, UK) diluted 1:25 in 1% BSA. Sections were then washed three times with PBS, incubated for 10 min with 1% formaldehyde in PBS, and washed again with PBS and dH_2_O three times each. Finally, sections were counterstained with uranyl acetate and lead citrate, and observed in a Jeol JEM 1010 transmission electron microscope.

### Morphometry of inner cell walls and SLs

In order to identify putative morphometric differences in the inner cell walls and/or SLs of the DH4079, DH12075, and DH3 lines, we used 15 randomly chosen images of microspore cultures of each line with at least one embryogenic microspore showing 1–2 cell divisions, taken under phase contrast at 100× from thin (1-µm) sections of resin-embedded samples. Morphometric measurements were performed using the Fiji distribution of the ImageJ open-source software (Schindelin *et al.*, 2012). For each image, at least 20 thicknesses were measured along the entire length of the SL, and 20 additional thicknesses along the inner cell walls. The average thickness (±SD) was calculated for each cell and wall type. Due to non-normality of some data, a Kruskal–Wallis test for median comparison followed by a Bonferroni test with a significance limit α<0.05 were performed.

### RNA isolation, cDNA synthesis, and quantitative real-time RT-PCR

Total RNA was isolated from rapeseed DH4079 microspores by phenol/chloroform extraction followed by precipitation with LiCl (Kay *et al.*, 1987) and stored at –80 °C. RNA (1–2 μg total RNA) was subjected to DNase treatment and reverse-transcription using a Maxima First-Strand cDNA Synthesis Kit (Thermo Fisher Scientific), according to the manufacturer’s instructions. To rule out the possibility of genomic DNA contamination, all cDNA sets were checked by control PCR reactions with aliquots of the same RNA subjected to the DNase treatment but not to reverse transcription. Gene expression analyses were carried out by real-time quantitative RT-PCR (qPCR) using a LightCycler 480 Real-Time PCR System (Roche). Each 23-μl reaction contained 3 μl of a 1:10 cDNA dilution, 10 μl of qPCR Green Master Mix (NZYTech), 6.4 μl of deionized water, and 0.8 μl of each primer pair (10 μM), as listed in Supplementary Table S1 at *JXB* online. The PCR program used was: 5 min incubation at 95 °C to activate the hot-start recombinant Taq DNA polymerase, and 45 cycles of 10 s at 95 °C, 10 s at 60 °C (annealing temperature), and 10 s at 72 °C, where the fluorescence signal was measured. The specificity of the PCR amplification procedure was checked with a heat dissociation protocol (from 65–100 °C) after the final PCR cycle. Relative quantification of specific mRNA levels was performed using the comparative 2^−ΔΔ*C*T^ method (Livak and Schmittgen, 2001). Expression values were normalized using the housekeeping gene *BnActin2* (Tan *et al.*, 2011). Experiments were repeated three times, with the threshold cycle (*C*_T_) determined in triplicate, using cDNAs that originated from three RNAs extracted from three different biological samples. Negative controls without cDNA were used in all PCR reactions. ANOVA was performed with a significance limit of α<0.05.

### Osmolality, viability, and embryogenic response

Three different DH4079 microspore cultures from the three bud-length groups described above were performed in parallel. From each culture, samples were taken at days 0, 3, and 7 after isolation. Viability was checked with fluorescein diacetate Rivas-Sendra *et al.*, (2017*a*), using 200-μl aliquots taken from the day-0 and day-3 cultures. To check the embryogenic response, 200-μl aliquots were taken from day-3 cultures and stained with DAPI, as described above. Counts were made of total and dividing microspores (defined as having two or more DAPI-stained nuclei and a cell wall between them) to calculate the percentage of divisions. For osmometry, cultures were collected in sterile 15-ml conical tubes and centrifuged (8000 rpm, 5 min). An aliquot of the culture medium was taken and analysed. Microspore pellets were transferred to 1.5-ml tubes and centrifuged (8000 rpm, 2 min). After removal of the supernatant, the pellets were frozen in liquid nitrogen and homogenized by five cycles of cold (–20 °C) and heat (55 °C) treatments, 10 min each. The homogenate was centrifuged (14 000 rpm, 5 min), the pellet was discarded and the supernatant was analysed. For both the culture medium and microspore contents, analysis of osmolality was performed with a 3320 Model Micro-Osmometer (Advanced Instruments, Inc.). Samples were directly injected and analysed following the manufacturer’s instructions. ANOVA) was performed with a significance limit of α<0.05.

## Results

### Microspores with different embryogenic responses develop SLs of different thickness

**Fig. 1.**
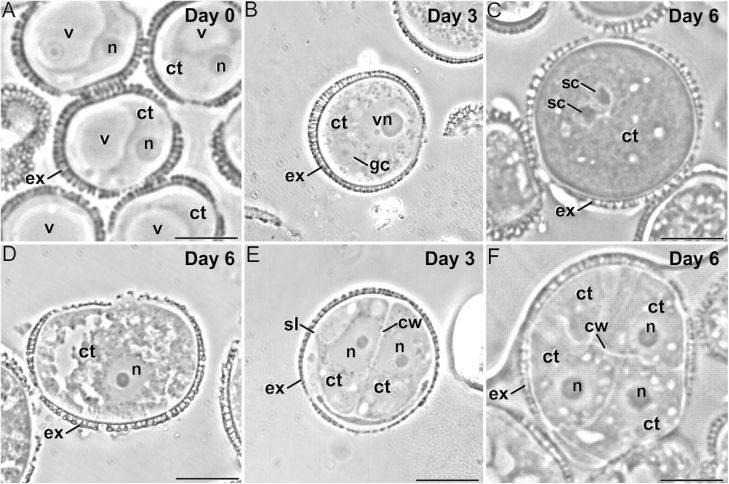
Development of microspore cultures of rapeseed line DH4079. (A) In the cultures, microspores are isolated and *in vitro* inoculated. Some microspores are not sensitive to the inductive treatment and follow a gametophytic-like program, becoming pollen-like structures (B) that enlarge and undergo a second pollen mitosis to become pollen-like grains (C). Others do not continue to develop and eventually die (D). However, microspores sensitive to the inductive treatment are reprogrammed towards embryogenesis, reabsorbing the large vacuole and dividing symmetrically. For example, in the highly embryogenic DH4079 line, 2–4-celled embryogenic structures can be observed just 3 d after inoculation (E), and multicellular structures (around eight cells) can be found in 6-d-old cultures (F). cw, cell wall; ct, cytoplasm; ex, exine; gc, generative cell; n, nucleus; sc, sperm cell; sl, subintinal layer; v, vacuole; vn, vegetative nucleus. Scale bars are 10 µm.

Microspore cultures are heterogeneous *in vitro* systems, where embryogenesis-committed microspores co-exist with dying, dead, or gametophytic-like structures (Fig. 1). Although all conformed with this general scheme, the cultured microspores of different genotypes showed notable differences. Besides their different embryogenic competence, the rapeseed DH4079 embryogenic microspores seemed to develop SLs that were visually thicker than the rapeseed DH12075 and eggplant DH36 (Supplementary Fig. S1). To confirm this, we measured the thickness of the SLs and first inner walls (Supplementary Fig. S2) and found that DH4079 microspores had a mean SL thickness (0.77±0.38 µm) that was significantly greater than those of DH12075 and DH36 (0.46±0.18 µm and 0.43±0.16 µm, respectively). In contrast, the three lines showed similar thicknesses for the inner walls (0.49±0.26, 0.48±0.21, and 0.46±0.22 µm for DH4079, DH12075, and DH36, respectively). We were thus able to demonstrate that the presence of SLs is inherent to embryogenic microspores, being not only present in the highly embryogenic rapeseed line DH4079, but also in recalcitrant lines and species. However, each genotype developed the SL to different extents. There appeared a correlation between embryogenic competence and SL thickness, but not with inner wall thickness, suggesting that the building of each wall might be regulated differently.

### The composition of SLs and inner cell walls of embryogenic microspores differs with genotype

**Fig. 2.**
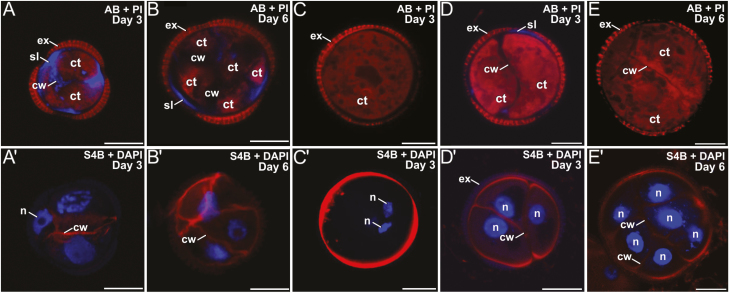
Callose and cellulose dynamics in microspore cultures of rapeseed lines DH4079 and DH12075. (A–E) Staining with aniline blue (AB, blue) and propidium iodide (PI, red) for visualization of callose and cytoplasm. (A′—E′) Staining with Pontamine Fast Scarlet (S4B, red) and DAPI (blue) for cellulose and nuclei. (A, A′) DH4079 3-d-old embryogenic microspore. (B, B′) DH4079 6-d-old multicellular embryogenic structure. (C, C′) DH4079 3-d-old pollen-like development in culture. (D, D′) DH12075 3-d-old embryogenic microspore. (E, E′) DH12075 6-d-old multicellular embryogenic structure. See text for further details. Ct, cytoplasm; cw, cell wall; ex, exine; n, nucleus; sl, subintinal layer. Scale bars are 10 µm.

**Fig. 3.**
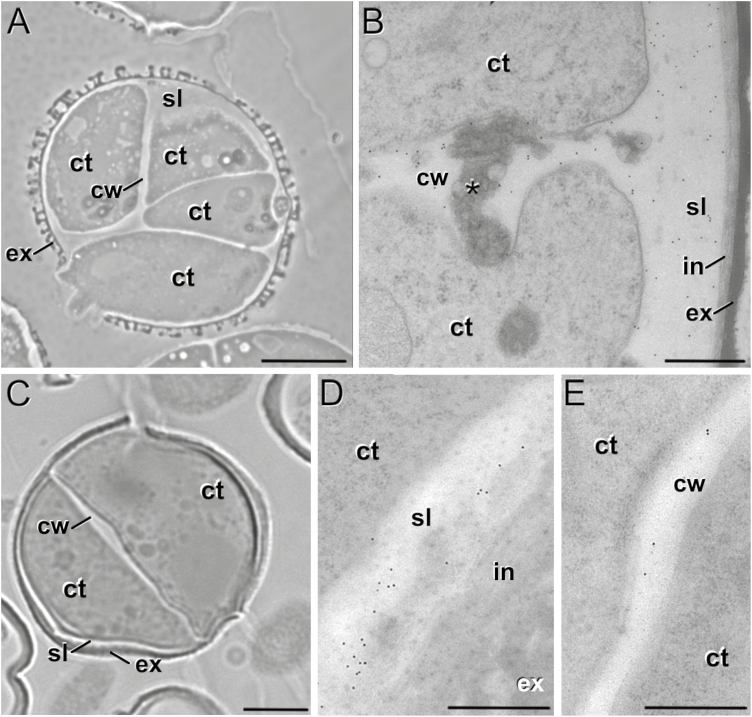
Anti-callose immunogold labeling of embryogenic microspores rapeseed line DH4079 and eggplant line DH36. (A) Rapeseed DH4079 embryogenic microspore and (B) detail of the inner cell wall (cw) and subintinal layer (sl) showing abundant gold particles. The asterisk indicates deposits of excreted material. (C) Eggplant DH36 embryogenic microspore, and detail of (D) the SL showing abundant gold particles and (E) the inner cell wall showing very scarce, dispersed gold particles. Ct, cytoplasm; ex, exine; in, intine. Scale bars: (A, C) 10 µm, (B, D, E) 500 nm.

We studied the dynamics of callose and cellulose deposition in the walls of the three genotypes. DH4079 embryogenic microspores early in induction (day 3) presented AB-stained (callose-positive) walls and SLs (Fig. 2A). Anti-callose immunogold labeling confirmed their callosic nature, which was clearly distinct from the intine and continuous with the inner cell walls (Fig. 3A, B). However, we never observed a similar S4B (cellulose-positive) signal in their SLs (Fig. 2A′). Multicellular structures (around 6–8 cells) presented no callose in the inner walls, and only small callose deposits in thin SL residuals, generally only below apertures (Fig. 2B). At this stage, we observed continuous but irregularly thick cellulose walls surrounding all cells (Fig. 2B′), which indicated that the SL was almost dismantled and the callose of the inner walls was replaced by cellulose. Pollen-like structures showed no detectable callose staining at any stage (Fig. 2C), whereas S4B staining revealed a thick and intensely stained cellulose layer, probably corresponding to the intine (Fig. 2C′). In summary, callose was considerably more abundant than cellulose at the beginning of MDE development in the DH4079 line. However, in the transition from two to six cells, callose from the inner walls was progressively replaced by cellulose, and the callose-rich SL was progressively dismantled.

In the rapeseed DH12075 line, microspores with 2–3 cells presented callose staining in the SL. However, the the SL was thinner and callose was much less abundant than in DH4079 (compare Fig. 2A, D). The inner walls were practically devoid of callose. All cells were surrounded by a S4B-positive cellulose layer continuous with the inner walls (Fig. 2D′). The callose and cellulose pattern was very similar in multicellular structures, with an absence of callose (Fig. 2E) and a thin, continuous cellulose wall entirely surrounding all cells (Fig. 2E′). In the embryogenic microspores of the eggplant DH36 line, a clear SL was also found, distinct from the intine but irregular in thickness and continuous with the inner cell walls (Fig. 3C). Although thinner, it was ultrastructurally identical to the SLs of DH4079. Immunogold labeling with anti-callose antibodies revealed the presence of callose in the SLs and inner walls (Fig. 3D, E), but it was much less abundant than in DH4079. Together, these observations indicated that callose-rich SLs and inner walls were not exclusive in DH4079: they also occurred in the recalcitrant genotypes, but to notably lower extents.

### Intracellular Ca^2+^ signals in embryogenic microspores differ with genotype

**Fig. 4.**
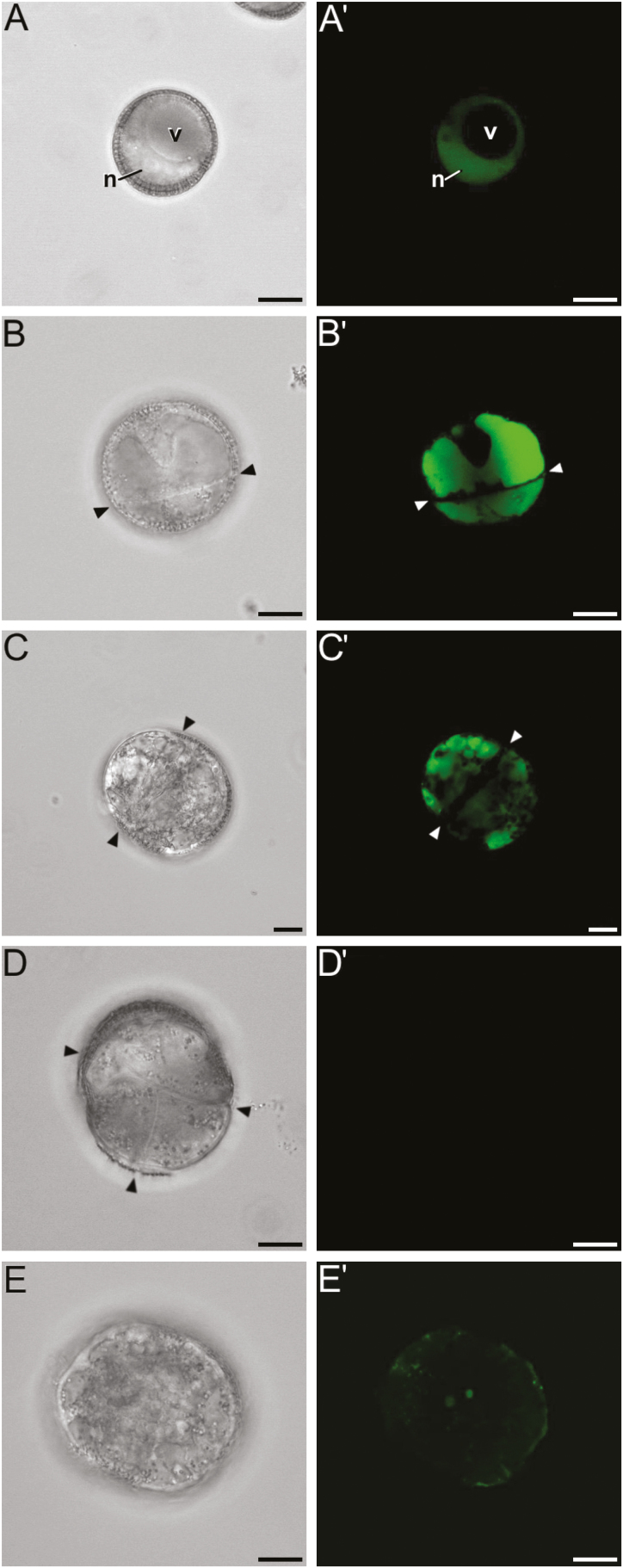
Dynamics of Ca^2+^ in cultured microspores of rapeseed line DH4079. Phase contrast (A–F) and FluoForte fluorescent staining (A′–F′) pairs of images are shown. (A, A′) Freshly isolated microspore showing a nuclear–cytosolic Ca^2+^ signal. (B, B′) Newly divided embryogenic microspore with an intense nuclear–cytosolic and vacuolar Ca^2+^ signal. The arrowheads indicate inner cell walls. (C, C′) Embryogenic structure with a Ca^2+^ signal that is principally vacuolar and is reduced in the nucleus/cytosol. (D, D′) Multicellular embryogenic structure with almost no detectable Ca^2+^ signal. (E, E′) Pollen-like structure with a faint, punctate Ca^2+^ signal that is mostly peripheral and at the centrally located sperm-like nuclei. N, nucleus; v, vacuole. Scale bars are 10 μm.

Ca^2+^ is known to activate some callose synthases and to inhibit cellulose deposition (Verma and Hong, 2001), and it has recently been related to the induction of embryogenesis (Parra-Vega *et al.*, 2015). We studied intracellular levels by means of the Ca^2+^-specific FluoForte molecular probe. Freshly isolated DH4079 microspores (i.e. not yet induced) presented a clear nuclear–cytosolic Ca^2+^ signal, with no signal in the vacuole (Fig. 4A, A′). The Ca^2+^ signal increased dramatically in 2-celled embryogenic microspores, being also present in the vacuoles (Fig. 4B, B′). The signal then decreased progressively with development, becoming scarce in the cytosol and accumulating in vacuoles (Fig. 4C, C′), before eventually reaching undetectable levels in multicellular structures (Fig. 4D, D′). In contrast, pollen-like structures showed a completely different pattern (Fig. 4E, E′). Taking these results together with those for SL thickness and composition, it appeared that deposition of callose, but not cellulose, in the SL and inner walls of embryogenic microspores was paralleled by a rise in intracellular Ca^2+^ levels. Later in development, the removal of callose and the deposition of cellulose coincided with a dramatic decrease of intracellular Ca^2+^. In other words, there seemed to be an association, specific to microspores committed to embryogenesis, between intracellular Ca^2+^ levels, callose deposition, and cellulose inhibition.

In 2-celled embryogenic microspores of the rapeseed DH12075 line (Supplementary Fig. S3A, A′), the Ca^2+^ signal was markedly lower and nuclear–cytoplasmic, and was excluded from the vacuoles, whilst multicellular structures presented almost no detectable Ca^2+^ signal (Supplementary Fig. S3B, B′), similar to DH4079. Pollen-like structures presented a scarce, punctate signal (Supplementary Fig. S3C, C′). In the eggplant DH36 microspores, the Ca^2+^ levels appeared to be between those of DH4079 and DH12075. Freshly isolated microspores presented a nuclear–cytosolic Ca^2+^ signal, excluded from the vacuole (Supplementary Fig. S4A, A′). The newly divided 2-celled microspores presented a moderate nuclear–cytosolic Ca^2+^ signal, but a more intense signal in the vacuoles (Supplementary Fig. S4B, B′). However, the overall signal was lower than in DH4079. Since microspore embryogenesis in eggplant proceeds more slowly than in rapeseed, it is normal to observe enlarged but still bicellular structures in 6-d old cultures. At this stage, the Ca^2+^ signal became faint and was exclusively nuclear–cytosolic (Supplementary Fig. S4C, C′), and it became almost undetectable in multicellular structures (Supplementary Fig. S4D, D′). In summary, intracellular Ca^2+^ levels increased transiently upon induction, but to different extents in the three genotypes. The increase was higher in the responsive genotype, which was where thicker SLs were developed and more callose accumulated.

### Caffeine reduces deposition of callose and cellulose, and prevents cell wall formation

**Fig. 5.**
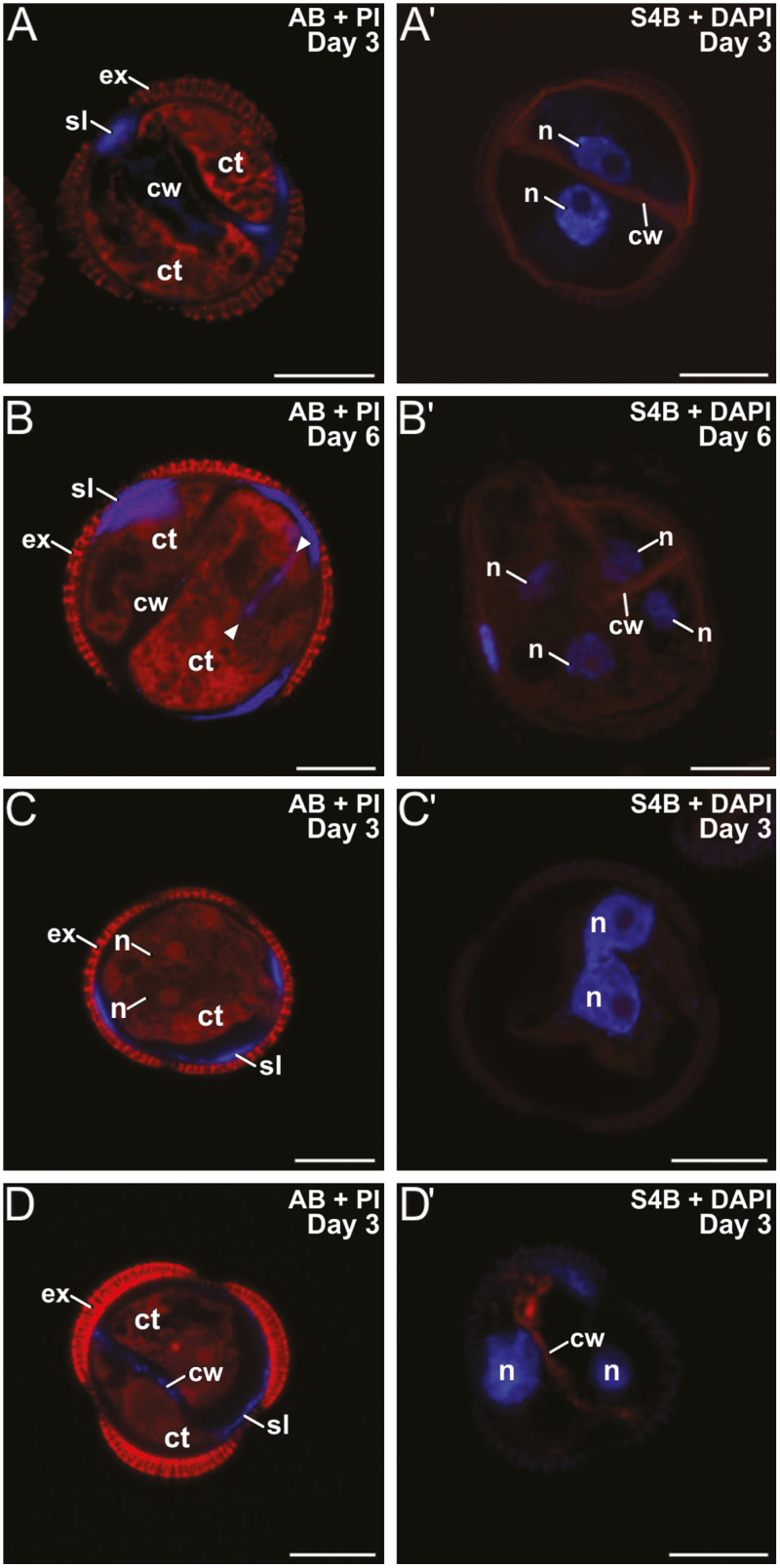
Microspores of rapeseed line DH4079 treated with caffeine. Paired images are shown of staining with aniline blue (AB) for callose and propidium iodide (PI) for cytoplasm, and with S4B for cellulose (red) and DAPI. Embryogenic microspores treated with 1 mM caffeine at (A, A′) 3 d old and (B, B′) 6 d old. The arrowheads indicate inner cell wall fragments. (C, C′) Embryogenic microspores treated with 10 mM caffeine at 3 d old. Note the presence of binucleated cells without inner walls and with two closely apposed (C) or even fused nuclei (C′). (D, D′): Examples of the few 3-d-old 2-celled embryogenic microspores treated with 10 mM caffeine that developed inner walls. ct, cytoplasm; cw, cell wall; ex, exine; n, nucleus; sl, subintinal layer. Scale bars are 10 µm.

The results presented above suggested a link, specific for microspores committed to embryogenesis, between increased Ca^2+^ levels, formation of callose-rich SLs and inner walls, and cellulose inhibition. We explored this link by altering intracellular Ca^2+^ levels using different chemicals and studying their effects on the formation of inner walls and SLs in the rapeseed DH4079 line. First, we added 1 mM or 10 mM caffeine to the microspore cultures. Three-day-old, 2-celled microspores treated with 1 mM caffeine presented thinner and more discontinuous inner cell walls with less callose than untreated microspores (Fig. 5A), whereas the SLs were similar to the untreated cultures. S4B staining showed thin but continuous cellulose walls around the cells (Fig. 5A′), as expected for conventional cytokinesis. In 6-d-old multicellular structures, callose and cellulose staining showed no notable differences with untreated cultures (Fig. 5B, B′), except for some ectopic wall fragments that were positive for AB staining (arrowheads in Fig. 5B). With 10 mM caffeine, embryogenic structures were unable to develop beyond the first division. Bi-nucleated cells without inner walls were often observed (Fig. 5C, C′). When present, cell walls were very thin, discontinuous, fragmented, and faintly stained for both callose and cellulose (Fig. 5D, D′). However, the effect of 10 mM caffeine was less severe on SLs, where callose was present even in cells without inner walls, and cellulose staining was negative (Fig. 5C, C′). Cells at 6 d old were all dead. Together, these results showed a dose-dependent effect of caffeine on callose deposition. At low doses, a reduction in callose synthesis was accompanied by an increase in cellulose. At higher doses, caffeine prevented the formation of inner walls, while SLs were considerably less affected.

### Digitonin causes an increase in callose and a reduction in cellulose

**Fig. 6.**
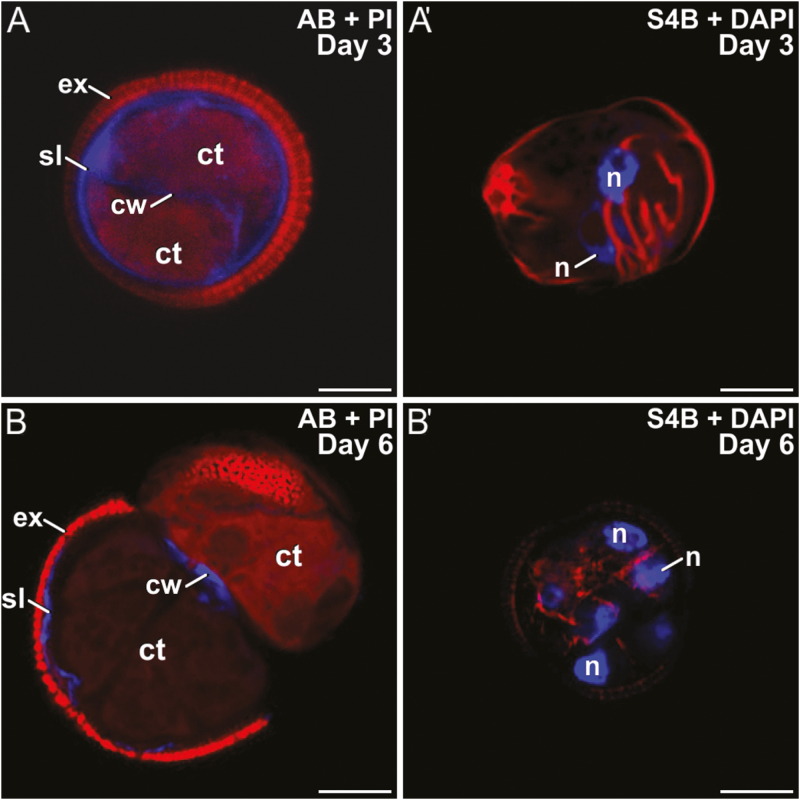
Microspores of rapeseed line DH4079 treated with 1 mg l^–1^ digitonin. Paired images are shown of staining with aniline blue (AB) for callose and propidium iodide (PI) for cytoplasm, and with S4B for cellulose (red) and DAPI. (A, A′) Embryogenic microspores at 3 d old. Note the presence of multiple ectopic, cytoplasmic wall fragments. (B, B′) Embryogenic microspores at 6 d old. ct, cytoplasm; cw, cell wall; ex, exine; n, nucleus; sl, subintinal layer. Scale bars are 10 µm.

We used digitonin to increase membrane fluidity and therefore the uptake of Ca^2+^. Rapeseed DH4079 microspores were cultured with 1, 5, 10, or 100 mg l^–1^ digitonin. With 5, 10, and 100 mg l^–1^, cells were all dead after 3 d of culture. However, those treated with 1 mg l^–1^ survived, and showed thicker callose walls that were more continuous and uniform than in untreated cells (Fig. 6A). Cellulose deposition was abnormal (Fig. 6A′). Later in development, multicellular structures presented thin, discontinuous, and fragmented callose deposits in the inner walls and SLs (Fig. 6B), and thin, incomplete cellulose deposits (Fig. 6B′). These results indicated that low concentrations of digitonin increased callose deposition and extended its presence over time, while cellulose deposition was severely altered or reduced.

### ES7 prevents callose deposition in the cell walls but not in the SLs and other callose-containing structures

**Fig. 7.**
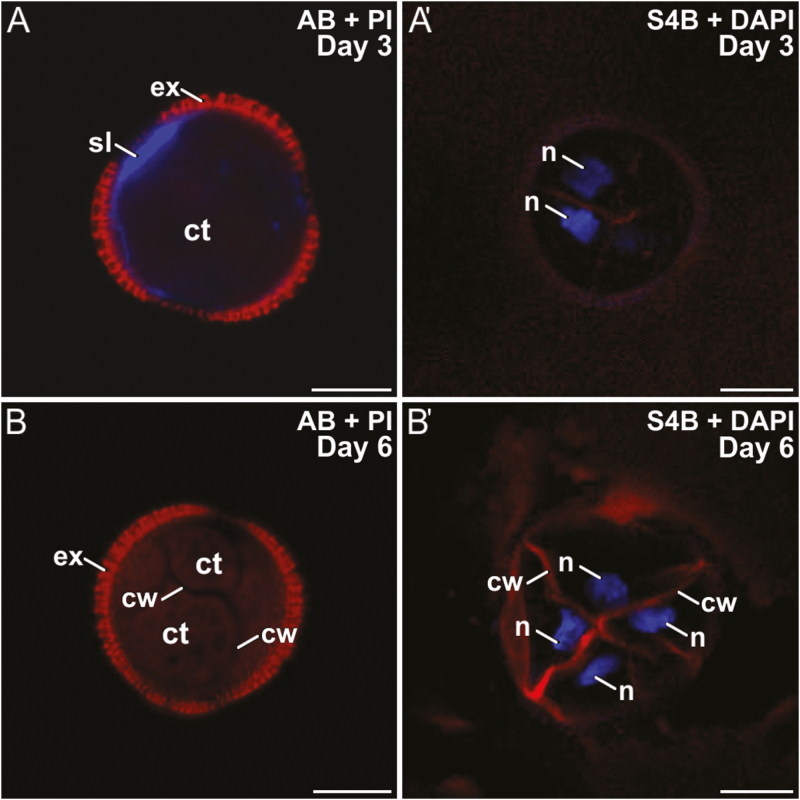
Microspores of rapeseed line DH4079 treated with 10 μM endosidin 7 (ES7) Paired images are shown of staining with aniline blue (AB) for callose and propidium iodide (PI) for cytoplasm, and with S4B for cellulose (red) and DAPI. Embryogenic microspores at (A, A′) 3 d old and (B, B′) 6 d old. ct, cytoplasm; cw, cell wall; ex, exine; n, nucleus; sl, subintinal layer. Scale bars are 10 µm.

Rapeseed DH4079 microspores were cultured with 10 μM or 25 μM ES7. As it was dissolved in DMSO, a control with DMSO alone was also performed. DMSO slightly reduced the efficiency of embryogenic induction, but no noticeable effects on polysaccharide deposition were observed. With 10 μM ES7, callose was found in the SL and beneath the apertures of 2-celled embryogenic microspores, but it was rarely or not detected in inner walls (Fig. 7A). Cellulose was found in some inner walls, but never in the SL (Fig. 7A′). In multicellular structures, only very small callose residuals, if any, were found beneath the intine, but never in inner walls (Fig. 7B). Cellulose deposition was unaffected (Fig. 7B′). Treatment with 25 μM ES7 had a stronger effect than 10 μM. Bi-nucleated cells, occasionally with fusing nuclei, were frequently observed (Supplementary Fig. S5A). Callose was present in the SL but not in inner walls (Supplementary Fig. S5B). Cellulose was faintly detected in the few inner walls formed and in SL fragments (Supplementary Fig. S5C). No further development was ever observed.

From these experiments, we deduced that application of ES7 affected microspores in a dose-dependent manner. At low concentrations, it strongly reduced callose deposition in inner walls, while cellulose deposition was slightly increased. At higher concentrations, it clearly blocked callose deposition in the cell plate, and therefore cells failed to divide. However, the SL remained unaffected and developed normally. These results suggested that while somatic-type cytokinesis was severely affected by ES7, callose deposition associated with SL formation was not negatively affected, which confirms the specific role of ES7 in somatic-type cytokinesis, and reinforces the notion that callose deposition in inner walls and SLs are different processes, differently affected by stimuli and inhibitors.

### Callose synthases are differentially expressed during microspore embryogenesis

**Fig. 8.**
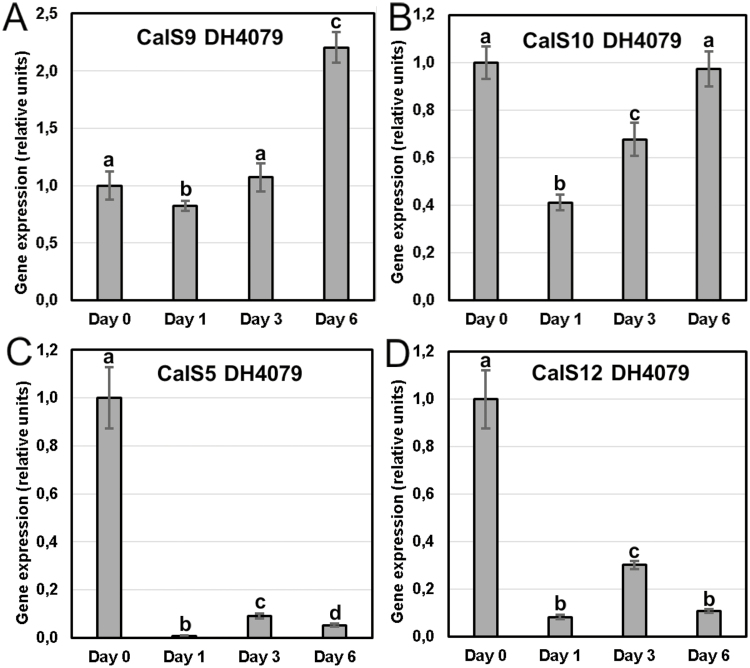
qPCR analysis of expression of callose synthase genes at different stages of microspore culture progression. (A) *CalS9/GSL10*, (B) *CalS10/GSL8*, (C) *CalS5/GSL2*, and (D) *CalS12/GSL5*. Expression is relative to the housekeeping gene *BnActin2* and in all cases the initial values (day 0) are normalized to zero. Data are means (±SD) of three replicates. Different letters indicate significant differences as determined by ANOVA (α≤0.05).

In order to shed light on the CalS complexes that could be involved in callose deposition during the formation of the unusual inner cell walls and SLs of the first divisions of embryogenic microspores, we analysed the expression of *CalS9/GSL10*, *CalS10/GSL8*, *CalS5/GSL2*, and *CalS12/GSL5* by qPCR during the initial stages of microspore embryogenesis in the rapeseed DH4079 line (Fig. 8). For all genes, we normalized the relative expression at day 0 (after microspore isolation but before induction) to a value of 1. Taking this value as the reference, all genes showed a significant decrease of their expression upon exposure to the inductive heat-shock treatment; however, their patterns were notably different. *CalS9/GSL10* presented a slight decrease at day 1, but then its expression increased steadily to a maximum at day 6, which was higher than at day 0 (Fig. 8A). *CalS10/GSL8* presented a very similar pattern, but the decrease upon exposure to heat shock was more pronounced (Fig. 8B). In contrast, *CalS5/GSL2* (Fig. 8C) and *CalS12/GSL5* (Fig. 8D) showed a remarkably different pattern, with a dramatic decrease at day 1 (100-fold for *CalS5/GSL2* and 10-fold for *CalS12/GSL5*) and then a slight increase at day 3, followed by another decrease at day 6. For both genes, the expression levels were always considerably below those of day 0. Together, these results demonstrated a clear association between exposure to heat shock and a decrease in the expression of the genes. Two expression patterns could be found: a sustained increase in gene expression for *CalS9/GSL10* and *CalS10/GSL8*, and a low-level for *CalS5/GSL2* and *CalS12/GSL5* with a peak at day 3.

### Protection against osmotic imbalance is different in embryogenic and non-embryogenic microspores

**Fig. 9.**
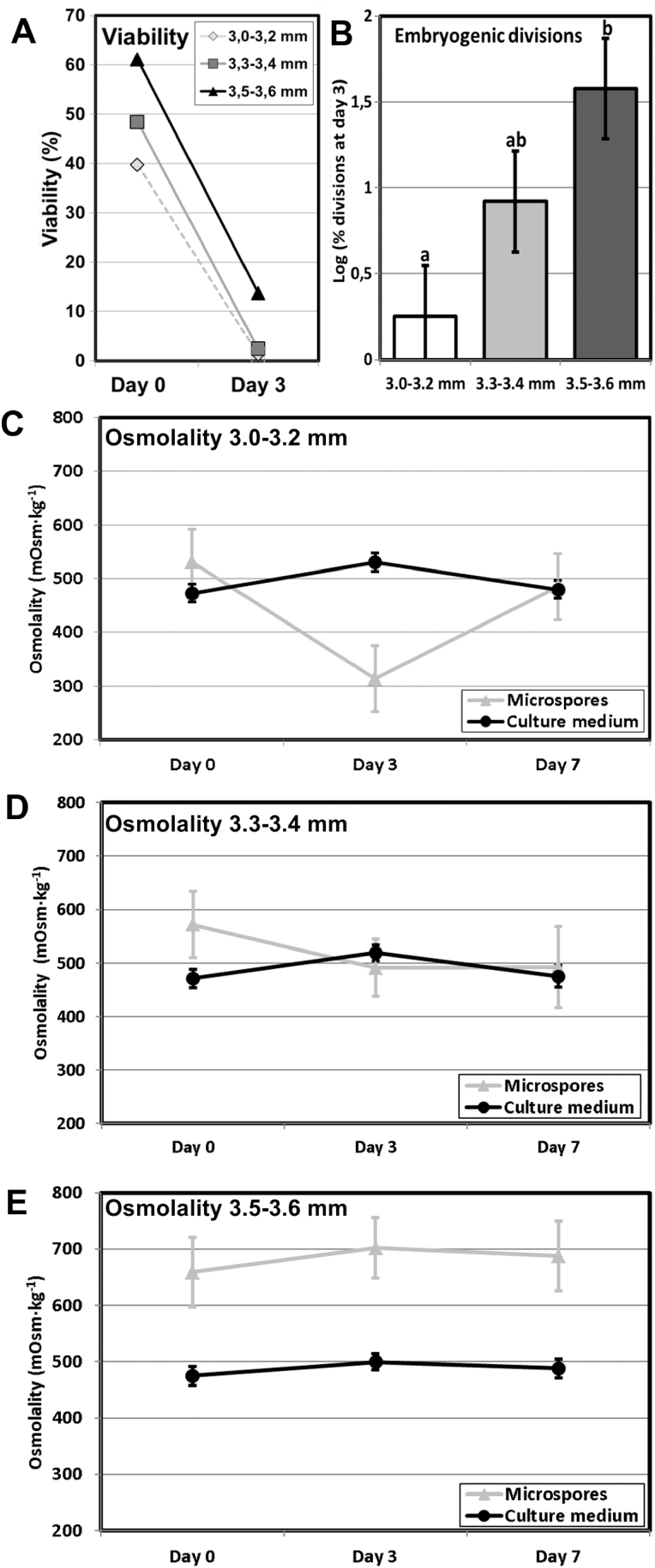
Viability, embryogenic response, and osmolality of microspores of rapeseed line DH4079 from bud-length groups of 3.0–3.2, 3.3–3.4, and 3.5–3.6 mm. (A) Viability of cultured microspores from the three groups at day 0 and day 3, as estimated by fluorescein diacetate (FDA) staining and expressed as percentage of living (FDA-positive) microspores. (B) Embryogenic response expressed as the logarithm of the percentage of dividing embryogenic cells at day 3. Data are means (±SD) of three replicates. (C–): Microspore and culture medium osmolality in cultures from the 3.0–3.2 mm (C), 3.3–3.4 mm (D), and 3.5–3.6 mm (E) bud-length groups, expressed as mOsm kg^−1^ at days 0, 3, and 7 after culture initiation. Data are means (±SD) of three replicates.

Our next goal was to shed light on the functional role of the SL. We designed an experiment to track the changes in viability, embryogenic response, and osmolality of the embryogenic microspores and the culture medium at different stages. To exclude the possible influences of genotype and culture conditions, instead of comparing different lines, we used microspores/pollen from the rapeseed DH4079 line isolated from the same donor plants exposed to identical culture conditions, but at different developmental stages. We isolated microspores from buds at 3.0–3.2, 3.3–3.4, and 3.5–3.6 mm, which contained mixed populations with a predominance of young microspores in the 3.0–3.2 mm group, mid-vacuolate microspores in the 3.3–3.4 mm group, and vacuolate microspores/young pollen grains in the 3.5–3.6 mm group. The viability of the three groups varied around 40–60% at day 0 and showed a dramatic decrease at day 3 (Fig. 9A). The 3.0–3.2 mm group showed ~0% viability, whilst that of the 3.3–3.4 mm was ~2%. In contrast, the 3.5–3.6 mm group showed a notably higher viability of 14%. As expected according to their composition, the most responsive group was 3.5–3.6 mm (Fig. 9B). Indeed, we found a high correlation between viability and divisions at day 3 (Pearson’s *r*=0.8443; *P*=0.0003). We measured the internal and medium osmolalities of the cultures at day 0 (just after isolation), day 3, and day 7. The osmolality of the medium remained nearly constant throughout (Fig. 9C–E), and the initial osmolality of the microspores of all the groups was remarkably similar, and always above that of the medium. However, different patterns were observed for the groups as the culture progressed. At day 3, the 3.0–3.2 mm and 3.3–3.4 mm groups dropped to values lower than at day 0, and then converged with those of culture medium again at day 7. In contrast, the pattern for the 3.5–3.6 mm group was completely different, with the nternal osmolality always being significantly higher than that of the medium and all individual values and internal-versus-medium differences being constant at all the measurement times. In summary, the bud-length group with highest viability and embryogenic response was also the only group where the microspores kept their osmolality values constant, and always above those of culture medium. This was the group containing a majority of microspores at the embryogenesis-inducible stages. We had shown that, upon induction, these were the only microspores to develop a callose-rich SL. In other words, we found a relationship between viability, embryogenic response (and subsequent formation of a callose-rich SL), and protection against an osmotically imbalanced medium.

## Discussion

### Callose deposition, cellulose inhibition, and intracellular Ca^2+^ levels are linked, and are related to genotype-specific embryogenic competence

In somatic-type cytokinesis, proper callose deposition is needed for subsequent deposition of cellulose. The high levels of Ca^2+^ needed for callose synthesis prevent premature cellulose synthesis (Verma, 2001), and when the levels decrease, callose synthesis stops and cellulose synthesis starts. Our experiments with digitonin showed that increases in callose deposition in the rapeseed line DH4079 were accompanied by a reduction in cellulose deposition (Fig. 6). In parallel, experiments with a low concentration of caffeine showed that limiting the level of Ca^2+^ reduced callose deposition and led to premature and increased cellulose deposition (Fig. 5). The results with higher doses of caffeine and with ES7 (Fig. 7) showed that when callose deposition was completely inhibited, cellulose was unable to be deposited. This indicated that, as in conventional somatic-type cytokinesis, the presence of a preliminary callose layer was needed as a scaffold to stabilize the cell plate and for proper cellulose deposition (Verma, 2001). However, callose must be removed to allow for cellulose deposition. Therefore, callose and cellulose synthesis during the first stages of microspore embryogenesis should be related but incompatible processes. As expected, our digitonin experiments (Fig. 6) demonstrated an increased initial deposition of callose and its persistence over time. These results confirmed the link between increased Ca^2+^ levels and callose deposition. Moreover, caffeine-induced reduction of intracellular Ca^2+^ (Fig. 5) blocked callose deposition in the cell plate, prevented cell division, and eventually caused cell death. These results indicated that Ca^2+^ plays a regulatory role over callose deposition in the cell plate that is essential for the proper initiation of microspore embryogenesis and for its success.

We also identified the same relationship between Ca^2+^ and microspore embryogenesis in the rapeseed line DH12075 and the eggplant line DH36, two recalcitrant lines that correspondingly experienced lower increases in Ca^2+^, lower callose deposition, and inhibition of cellulose compared to DH4079. In essence, we established a direct relationship between a high embryogenic response and transient intracellular Ca^2+^ increases, massive callose deposition, and cellulose inhibition. Conversely, the recalcitrant genotypes presented opposing phenotypes, the extent of which was proportional to the level of their recalcitrance.

### Callose deposition in the cell wall and the SL are differently regulated processes

The different timing and patterns of callose deposition at different locations, and in response to different stimuli even when occurring at the same time, indicated that a local activation of callose synthases was present in the microspores/pollen at the stages studied (Figs 1–3). Our results consistently showed that callose deposition was different in newly formed inner walls and subintinal layers (SLs). Callose is deposited in the SL even before the first embryogenic division (Parra-Vega *et al.*, 2015). While callose accumulated in the inner walls of only DH4079, it accumulated in the SL of DH4079 and, to lower extents, DH36 and DH12075, which were thinner than in DH4079 (Supplementary Fig. S2). Experiments with caffeine (Fig. 5) showed that reduced intracellular Ca^2+^ led to reduced callose deposition in the inner walls, but the callose content of the SL was less affected, irrespective of the caffeine dose. In parallel, cytokinesis was disrupted by ES7 (Fig. 7), whereas the effect on SL formation was much less noticeable. These results indicate that callose deposition in the inner walls and SLs is differently regulated and with different timing.

### Involvement of *CalS9/GSL10* and *CalS10/GSL8* in callose deposition in the first inner walls

The expression of the four CalS genes that we studied decreased immediately after heat shock (Fig. 8). This was not surprising since heat shock is known to severely affect transcription and translation in general (Alexandre *et al.*, 2014; Mahat *et al.*, 2016) and in particular upon induction of rapeseed microspore embryogenesis (Cordewener *et al.*, 2000; Joosen *et al.*, 2007; Malik *et al.*, 2007). After the initial decrease, we identified two gene expression patterns: a sustained increase for *CalS9/GSL10* and *CalS10/GSL8*, and a peak at day 3 for *CalS5/GSL2* and *CalS12/GSL5*.

CalS9/GSL10 and CalS10/GSL8 are responsible for the callose-rich wall of generative cells, so they are already present in the cell plate or ready for cell plate delivery when microspores change their fate. CalS10/GSL8 is also involved in somatic-type cytokinesis (Chen *et al.*, 2009), being functionally redundant with the cell plate-specific CalS1/GSL6 (Hong *et al.*, 2001). The steady increase in expression of these two genes must only have come from the dividing cells in the cultures (embryogenic microspores), and it was consistent with the initial massive deposition in the inner walls (Figs. 2, 3), and with the increased need for callose in cell plates as more cells divided and new walls were formed in MDEs. Therefore, we propose that *CalS9/GSL10* and *CalS10/GSL8* may be responsible for callose deposition in the first walls formed in embryogenic microspores. The strong influence of Ca^2+^ on callose deposition in the inner walls, as revealed by our experiments with caffeine, digitonin, and ES7 (Figs 5–7), suggests that at least one of these genes, but possibly both, is regulated by Ca^2+^, either directly or indirectly.

### Involvement of *CalS5/GSL2* and *CalS12/GSL5* in the formation of the SL

After a drastic decrease upon exposure to heat shock, expression of *CalS5/GSL2* and *CalS12/GSL5* increased significantly at day 3, and then dropped down again (Fig. 8). Interestingly, this increase–decrease dynamic paralleled that of callose deposition at the SL (Fig. 2). No new SLs formed in multicellular structures at 6 d after the heat shock. These results suggest that CalS5/GSL2 and CalS12/GSL5 may be responsible for callose deposition in the SLs. Other indirect evidence reinforces this notion. The spatial pattern of callose deposition in the SL resembled the continuous callose layer deposited by CalS12/GSL5 between the plasma membrane and the cell wall of Arabidopsis epidermal cells as a defense barrier in response to the penetration of fungal haustoria (Jacobs *et al.*, 2003). Given the coincidences between microspore embryogenesis and pathogen response (see Introduction), exposure to heat stress and increases in Ca^2+^ may activate Ca^2+^-dependent CalS12/GSL5 during the first stages of embryogenesis. On the other hand, CalS5/GSL2 is responsible for the thin callose layer deposited around young microspores to serve as a scaffold for exine formation (Dong *et al.*, 2005). It is therefore present in microspores and could be activated by factors as yet unknown but, in principle, different from Ca^2+^. Our experiments repeatedly showed that changes in intracellular Ca^2+^ altered callose deposition in the inner walls, but the SL was much less (or not) affected (Fig. 4, Supplementary Figs S3, S4). Even in the recalcitrant genotypes there was always a basal level of Ca^2+^-independent callose deposition. Thus, we propose that CalS5/GSL2 and CalS12/GSL5 may be transiently activated during the first stages of embryogenesis induction to make callose deposition at the SL a robust event that is independent from cytokinesis.

### SLs act as osmoprotective barriers in embryogenic microspores

Upon exposure to heat stress and culturing in hypotonic medium for 3 d, the only bud-length group that kept osmolality values unchanged was 3.5–3.6 mm, which was also the group that showed the highest viability and embryogenic response (Fig. 9). The most plausible explanation for this is that the combination of heat and hypotonic stress killed almost all the cells in the three bud-length groups, with the exception of those induced to embryogenesis, which were more abundant in the 3.5–3.6 mm group and which presented a callose-rich SL (Figs 2, 3). Thus, it is reasonable to propose that SLs have a protective effect in embryogenic microspores. The convergence of internal and external osmolalities in the groups with smaller bud lengths may be an indication that upon heat shock, destabilized plasma membranes of the non-induced cells may not be able to prevent massive water uptake from the hypotonic medium, and hence these cells burst and die. In contrast, the heat shock-altered membranes of the embryogenic cells are surrounded by SLs and are therefore more protected from water uptake.

Microspores/pollen of the 3.5–3.6 mm group had almost fully developed intines and exines (data not shown), which might also have contributed to protection and we cannot rule this out as a possibility. However, the protection could not have come exclusively from naturally formed walls, since the equivalent DH12075 microspores/pollen at the same developmental stage, exposed to the same induction and culture conditions but which developed thinner SLs (Supplementary Fig. S2), were extremely recalcitrant to induction and nearly all died soon after heat shock (data not shown). Indeed, we demonstrated in DH4079, DH12075, and DH36 that embryogenic competence was linked to SL thickness and callose deposition. Together, these results indicated that the observed osmoprotective effect must to a large extent have come from the callose-rich SLs. This role is not surprising if we consider the chemical properties of callose, a self-aggregating polymer that forms dense, insoluble, highly hydrated and semi-permeable gels that are known to reduce water permeability (Vithanage *et al.*, 1980; Bhalla and Slattery, 1984). This makes them ideal as physiochemical barriers (Albersheim *et al.*, 2011).

### The formation of a callose-rich wall is a protective response common to a wide variety of different morphogenic processes in different species and genetic backgrounds

In plant biology, there is a wealth of examples of transient callose synthesis triggered by heat stress, both under experimental and field conditions (reviewed by Stass and Horst, 2009). Callose walls have also been described at the onset of other natural and *in vitro* morphogenic processes. For example, to prevent osmotic stress during male and female meiosis (Zhang *et al.*, 2002; Abramova *et al.*, 2003), during induction of somatic embryogenesis (Maheswaran and Williams, 1985; Dubois *et al.*, 1991; You *et al.*, 2006), during *in vitro*-induced organogenesis (Fortes *et al.*, 2002), in the newly formed cell wall of carrot protoplasts (Stass and Horst, 2009), and during *in vitro* culture of BY-2 cells, where callose walls are associated with increased viability through tolerance to osmotic stress in imbalanced media (Xie *et al.*, 2012). Other examples relate to male gamete formation (Töller *et al.*, 2008) and zygotic embryogenesis (Jensen, 1968; Williams *et al.*, 1984). Thus, the main role of these special walls relates to increased viability in artificial, osmotically imbalanced *in vitro* environments. Recalcitrant genotypes such as DH36 and DH12075, with thinner SLs and very low callose deposition, would be less protected against osmotic stress, and therefore much less viable. However, SLs may have additional roles. Given that in all these processes the synthesis of a callose barrier by a cell is related to a change in its developmental fate, it seems reasonable to propose an additional role in the isolation of cells from molecular cues, thereby allowing for the expression of new developmental programs without external interference. Thus, formation of callose-rich protective layers appears as a widely adopted mechanism to isolate specific cells from the physiochemical influence of the surrounding environment.

## Concluding remarks

**Fig. 10.**
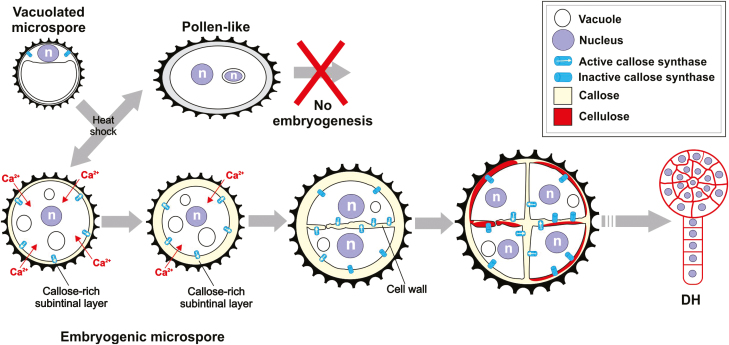
Proposed model for the formation of callose-rich inner walls and the osmoprotective subintinal layer (SL) barrier. In non-responding microspores, heat shock may cause arrest, death, or have no effect, allowing for the development of non-embryogenic, pollen-like structures. In responding microspores, heat shock may generate an increased Ca^2+^ influx and may activate different sets of callose synthases to massively synthesize callose, first at the plasma membrane to generate the SL and then in the inner walls. After some divisions, and long after the effects of the heat shock, callose would be progressively degraded. This callose removal would allow for normal cellulose deposition as in conventional, somatic-type cytokinesis. Progressive adoption of this conventional cytokinetic pattern would allow for proper cell proliferation and progression of the microspore-derived embryo (MDE), giving rise to haploid or doubled-haploid MDEs. n, nucleus; DH: doubled-haploid MDE.

Our results point to a sequence of events beginning with the application of the stress treatment, which is differently perceived by microspores of different genotypes with different stress tolerance, and which generates different responses, as detailed in Fig. 10. In responsive microspores, the mechanism produces two types of callose-rich walls that significantly influence MDE development. Callose-rich inner walls are related to the ability of embryogenic microspores to undergo genome-doubling through nuclear fusion (Parra-Vega *et al.*, 2015). On the other hand, callose-rich SLs would isolate and protect microspores during the first culture stages, thereby increasing their viability and the chances of a successful developmental switch.

In summary, we have identified one of the causes of recalcitrance to embryogenesis induction: a reduced or absent protective wall. It remains unknown whether this is due to a reduced ability for Ca^2+^ mobilization or uptake, differences in Ca^2+^ signaling, different activity or Ca^2+^-dependence of CalSs, or a combination of these factors. In any case, it seems related to a more general protective mechanism, present in responsive genotypes and absent in recalcitrant ones. Dubas *et al.*, (2013) showed that upon exposure to heat shock, rapeseed DH4079 microspores increase their plasma membrane rigidity in order to compensate for the fluidization caused by the shock; recalcitrant genotypes have no such mechanism and therefore the embryogenic response is much lower. Similarly, an additional element of this mechanism of DH4079 (and not of recalcitrants) may be the development of an osmoprotective subintinal layer.

## Supplementary data

Supplementary data are available at *JXB* online.

Table S1. Primer pairs used for qPCR.

Fig. S1. Overview of microspore cultures.

Fig. S2. Morphometric analysis of inner wall and SL thickness.

Fig. S3. Dynamics of Ca^2+^ in cultured microspores of rapeseed line DH12075.

Fig. S4. Dynamics of Ca^2+^ in cultured microspores of eggplant line DH36.

Fig. S5. Microspores of rapeseed line DH4079 treated with 25 μM ES7.

Supplementary Figures S1-S5

## Author contributions

ARS performed the callose and cellulose detection assays, and all the treatments with caffeine, digitonin, and ES7; PCM conducted the electron microscopy studies, callose and cellulose detection assays in DH12075, and different microspore cultures; RP performed the qPCR analysis; CCF conducted all the assays for Ca^2+^ detection in the DH36 and DH12075 lines; ACS did the viability and osmometry assays; JMSS designed and directed the study and experiments, and wrote the paper.
