# Supplementary material for: Embryogenic competence of microspores is associated with their ability to form a callosic, osmoprotective subintinal layer
Source: J Exp Bot. 2019 Jan 31;70(4):1267–81. doi: 10.1093/jxb/ery458 (PMC6382338; doi:10.1093/jxb/ery458)

Supplementary data

Article title: Embryogenic competence of microspores is associated to their ability to form a callosic, osmoprotective subintinal layer.

Authors: Alba Rivas-Sendra, Patricia Corral-Martínez, Rosa Porcel, Carolina Camacho-Fernández, Antonio Calabuig-Serna and Jose M. Seguí-Simarro

The following Supporting Information is available for this article:

Table S1. Primer pairs used for qPCR.

Gene name	*Primer Sequence 5' to 3'*
BnActin2 Forward	ACGAGCTACCTGACGGACAAG
BnActin2 Reverse	GAGCGACGGCTGGAAGAGTA
BnCalS5 Forward	GTGGATTGAACTGGCCATCTG
BnCalS5 Reverse	GGTTTCTGACATTGTTTGCCTG
BnCalS9 Forward	GGAATGCTGATATGCTTG
BnCalS9 Reverse	GGCGGGATTGCTCATTAGC
BnCalS10 Forward	GGCGATTAGATACACTGAACATTTCC
BnCalS10 Reverse	AAGTGTGAGAACCAGATGCTCC
BnCalS12 Forward	TGCTACGGTTTGGATAATTGCT
BnCalS12 Reverse	TCAGGGACCAAGAAAGCCAC

Fig. S1: Overview of microspore cultures. General views of the DH4079 (A), DH12075 (B) and DH36 (C) cultured populations, 3 (A, B) and 6 (C) days after culture initiation. Arrows point to embryogenic microspores, much more frequent in DH4079. Bars: 50 µm.

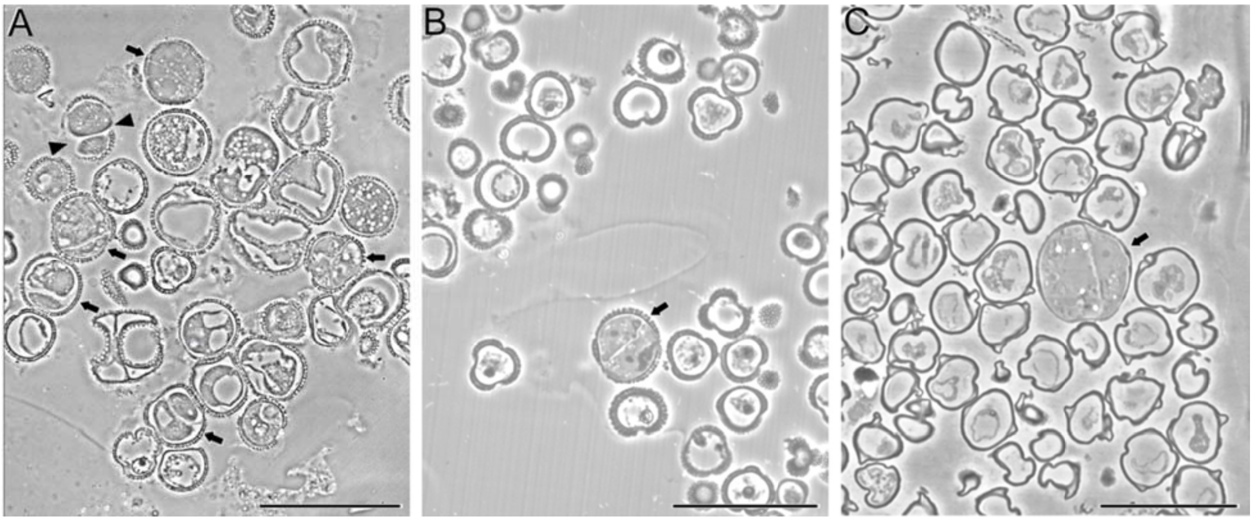

Fig. S2: Morphometric analysis of inner wall and SL thickness in the DH4079, DH12075 and DH36 lines. A: Average wall thickness comparison of the three lines Different letters indicate significant differences as revealed by the Kruskal-Wallis and Bonferroni tests with α≤0.05. B-D: Averages of all the measurements done for each of the 15 individual cells analyzed for DH4079 (B), DH12075 (C) and DH36 (D). Results are expressed as thickness ± S.D.

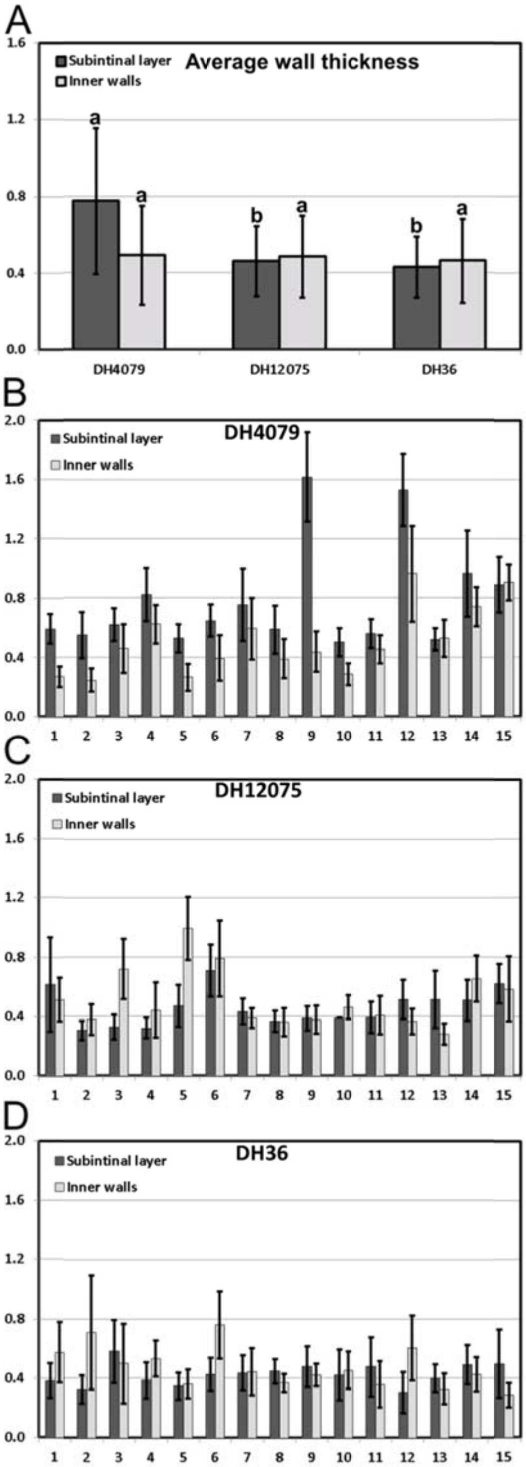

Fig. S3: Dynamics of Ca^{2+} in rapeseed DH12075 cultured microspores. Phase contrast (A-C) and FluoForte fluorescent staining (A'-C') pairs of pictures are shown. A, A': Just divided, embryogenic microspore with a moderate and principally nuclear-cytosolic (not vacuolar) Ca^{2+} signal. Arrowheads indicate inner cell walls. B, B': Multicellular embryogenic structure with almost no detectable Ca^{2+} signal. C, C': Pollen-like structure with a faint, punctate Ca^{2+} signal. ex: exine; n: nucleus; v: vacuole. Bars: 10 μm.

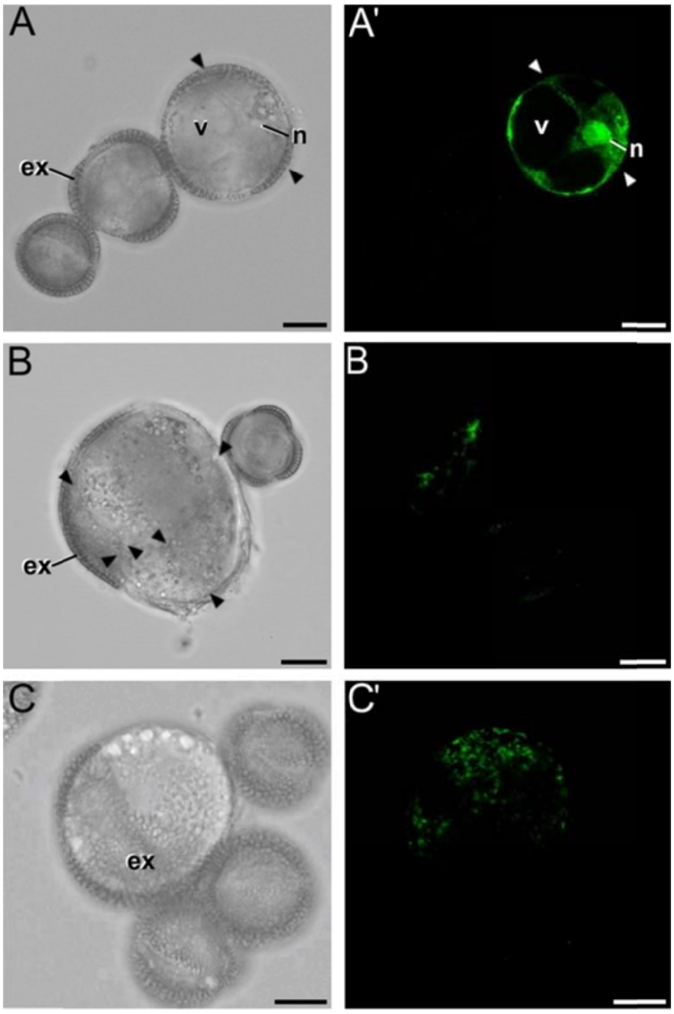

Fig. S4: Dynamics of Ca^{2+} in eggplant DH36 cultured microspores. Phase contrast (A-C) and FluoForte fluorescent staining (A'-C') pairs of pictures are shown. A, A': Freshly isolated microspore showing a faint nuclear-cytosolic (not vacuolar) Ca^{2+} signal. B, B': Just divided, embryogenic microspore with a moderate nuclear-cytosolic, but also vacuolar Ca^{2+} signal. Arrowheads indicate inner cell walls. C, C': Embryogenic structure with reduced nuclear-cytosolic and vacuolar Ca^{2+} signal. D, D': Multicellular embryogenic structure with almost no detectable Ca^{2+} signal. n: nucleus; v: vacuole. Bars: 10 μm.

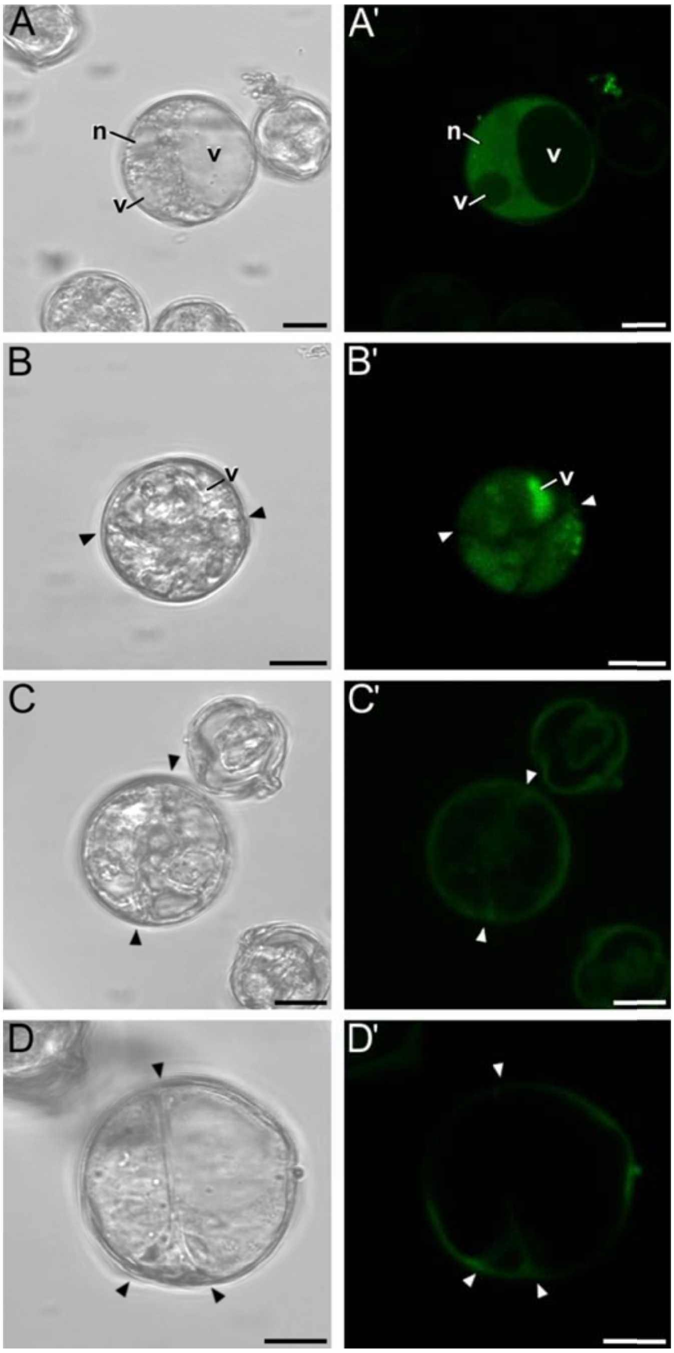

Fig. S5. Rapeseed DH4079 microspores treated with ES7 25 µM. A: DAPI (blue) stained 3-day old microspore showing two fusing nuclei. B: 3 day-old embryogenic microspore stained with aniline blue for callose (blue) and propidium iodide (red). Note that the red fluorescence of exine is due to autofluorescence, not to propidium iodide staining. C: 3-day old embryogenic microspore stained with S4B for cellulose (red) and DAPI (blue). cw: cell wall; ex: exine; n: nucleus; sl: subintinal layer. Bars: 10 µm.

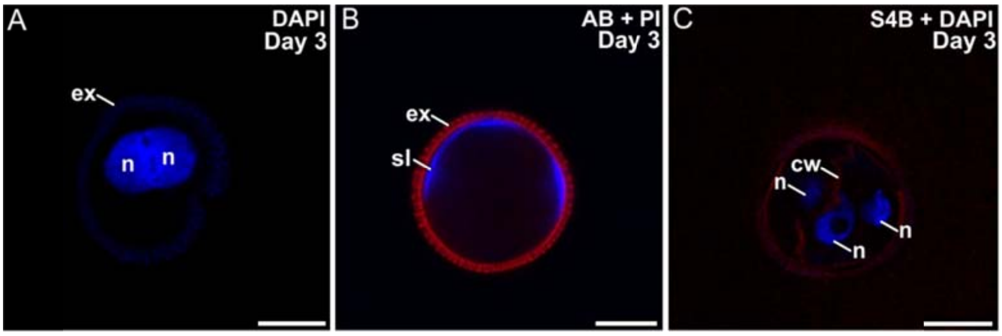

Supplement: Supplementary Figures S1-S5 [file ery458_suppl_supplementary-figures-s1-s5.pdf]
